# Perioperative Piperacillin/Tazobactam Reduces Early Onset SSI in Preirradiated Patients Undergoing Microvascular Head and Neck Reconstruction

**DOI:** 10.1002/hed.70151

**Published:** 2026-01-08

**Authors:** Johannes G. Schuderer, Florian Hoferer, Jonas Eichberger, Mathias Fiedler, André Gessner, Stilla Bauernfeind, Maximilian Gottsauner, Felix Nieberle, Michael Maurer, Johannes K. Meier, Torsten E. Reichert, Tobias Ettl

**Affiliations:** ^1^ Department of Oral and Maxillofacial Surgery University Hospital Regensburg Regensburg Germany; ^2^ Institute of Clinical Microbiology and Hygiene University Hospital Regensburg Regensburg Germany; ^3^ Department of Infection Prevention and Infectious Diseases University Hospital Regensburg Regensburg Germany

**Keywords:** head neck, microvascular reconstruction, osteonecrosis, perioperative antibiotic prophylaxis, SSI

## Abstract

**Background:**

Preirradiated patients undergoing microvascular head and neck reconstruction for tumor recurrence or osteoradionecrosis (ORN) face surgical site infection (SSI) rates exceeding 30%. The impact of perioperative extended‐spectrum antibiotic coverage remains unclear. This study hypothesizes that calculated prophylaxis with piperacillin/tazobactam reduces early‐onset SSI in this high‐risk population.

**Methods:**

From a microvascular reconstructed cohort, 161 with prior irradiation were retrospectively identified (tumor, *N* = 101; ORN, *N* = 60). SSI risk reduction was compared between patients receiving piperacillin/tazobactam (*N* = 39) and standard prophylaxis (*N* = 122).

**Results:**

With underlying SSI incidence of 36%, piperacillin/tazobactam showed a fourfold SSI risk reduction (HR 0.24; *p* = 0.002) across the entire cohort and a fivefold reduction in patients with bone resection (HR 0.17; *p* = 0.01). Subgroup analyses confirmed this effect with a sevenfold reduction in tumor patients (HR 0.14; *p* = 0.008) and a threefold reduction in ORN patients (HR 0.29; *p* = 0.04). Extended coverage did not significantly prolong antibiotic treatment times.

**Conclusion:**

In summary, calculated use of piperacillin/tazobactam in previously irradiated patients requiring microvascular reconstruction appears effective in reducing early‐onset SSI.

## 1 Introduction

Surgical site infections (SSI) are dreaded complications representing the most common cause for escalation or continuation of perioperatively antibiotic administration in microvascular reconstructive head and neck surgery. Reported incidence rates range from 5% to 15% in low‐risk patients to 33%–60% in irradiated high‐risk cohorts [1, 2, 3]. Scientific discussions have focused on explaining risk factors for the occurrence of SSI, establishing predictors including preoperative radiotherapy, poor ASA status, perioperative blood transfusion, and flap failure [4, 5, 6]. Due to Antibiotic Stewardship (ABS) efforts, attention has shifted defining the optimal duration of perioperative antibiotic prophylaxis (PAP) [3, 7]. Current systematic evidence indicates that prolonged PAP does not confer an advantage over shorter regimens (< 48 h) with respect to SSI rates [4, 8, 9]. However, these findings must be interpreted with caution since available studies are exclusively retrospective and often based on highly preselected cohorts.

Clinical experience further highlights another important trend: an increasing proportion of patients undergoing complex microvascular reconstructions are seen with prior oncologic treatment. These patients often require surgery in the context of tumor recurrence, salvage procedures following primary radio/chemotherapy or secondary reconstructions for osteoradionecrosis (ORN) [10, 11, 12]. Patients with prior oncologic therapies are frequently colonized with multidrug‐resistant Gram‐negative organisms, which may be selected and promoted by inadequate or unnecessarily prolonged prophylaxis, thereby directly pushing SSI incidences and highlighting the question for adequate antibiotic calculation [13, 14, 15].

Consistent with general osteomyelitis management, jaw bone infections are typically treated with a four‐ to six‐week course of antibiotic therapy following bone resection [16, 17]. Patients presenting SSI undergone salvage tumor surgery or resection of ORN with immediate microsurgical reconstruction do not fit well into this scheme. Intraoperative biopsy collection for microbiological culture‐guided antibiotic therapy is being discussed as a potential alternative to prolonged intravenous regimens, allowing for shorter treatment courses in patients with ORN [13, 18]. In literature, the duration of perioperative antibiotic application in microvascular reconstructions in previously irradiated patients varies considerably. Depending on risk stratification, flap type, defect size, and bone reconstruction, antibiotic courses may be extended from 24 h to up to 10 days postoperatively [19, 20]. In all cases, the primary objective is the uneventful integration of the microsurgical graft to enable long‐term functional rehabilitation.

Yet it is scientifically unaddressed whether a calculated perioperative antibiotic coverage with extended‐spectrum coverage provides clinical benefit in patients undergoing microvascular reconstruction after radiotherapy. This retrospective study hypothesizes that calculated perioperative piperacillin/tazobactam administration reduces the incidence of early‐onset SSIs in pre‐irradiated patients.

## 2 Material and Methods

The medical records of patients who underwent microvascular head and neck reconstruction in our department between 2016 and 2025 were reviewed. Patients with incomplete medical history or documentation were excluded from the analysis. Of these, patients who had received preoperative radiotherapy were included in the final statistical evaluation.

Perioperative antibiotic administration was defined from the first intravenous intraoperative dose until cessation. Standard regimens followed institutional SOPs, with intravenous amoxicillin/clavulanic acid as first‐line therapy and cephalosporins or lincosamide in case of allergy. In case of prior irradiation, piperacillin/tazobactam was set as preferred perioperative prophylaxis. Data analysis focused on type of agent, duration, and escalation. Antibiotic‐related adverse events were not assessed.

SSI were defined according to the Centers for Disease Control and Prevention (CDC) criteria [21]. In suspected SSI cases, postoperative microbiological swabs were obtained and processed following EUCAST standards for susceptibility testing [22]. Other nosocomial infections (e.g., pneumonia) were recorded from discharge documentation, limited to events during hospitalization [23]. If clinically indicated, antibiotic treatment was escalated due to antibiogram results [22].

The following variables were retrospectively assessed: demographic data (age, sex), diagnosis, ASA score, length of ICU and normal ward stay, defect site, flap type, bone resection, neck dissection, preoperative radiotherapy. Flap‐related outcomes included overall success, local complications (arterial/venous congestion requiring revision), and flap loss, defined as partial or complete necrosis impairing reconstruction.

## 3 Statistical Analysis

Univariate associations were tested using Fisher's exact test, chi‐square test, and Student's *t* test as appropriate. Clinically relevant factors were then included in binary logistic regression models (maximum likelihood estimation), reporting regression coefficients (*B*) and odds ratios (ORs) with 95% confidence intervals (CIs). A Cox proportional hazards regression model was applied to estimate hazard ratios (HRs) and 95% CIs for the association between prior piperacillin/tazobactam exposure and time to event (SSI). Model fit was evaluated by ANOVA; effect sizes were expressed as *f* [2], with values ≥ 0.35 indicating a strong effect. A significance threshold of *p* ≤ 0.05 was applied. Analyses were conducted using SPSS v29.0 (IBM Corp.). Regarding Table 1 percentages for binary variables refer to the total study population (SSI). In cases of multiple categories, percentages are reported relative to the comparison group, as in a cross‐tabulation (chi‐square test).

**TABLE 1:** Clinical characteristics.

Variable		Total (*N* = 161)	SSI yes (*N* = 58)	*p*
Sex				NS
	Male	98 (60.9%)	36 (62.1%)	
	Female	63 (39.1%)	22 (37.9%)	
Age	Years	64.7 ± 10.4	64.3 ± 8.7	NS
Diagnosis				0.02
	Tumor	101 (62.7%)	30 (51.7%)	
	ORN	60 (37.3%)	28 (48.3%)	
Flap				< 0.001
	FIB	67 (41.6%)	37 (63.8%)	
	RFF	41 (25.5%)	8 (13.8%)	
	ALT	29 (18%)	7 (12%)	
	Double	4 (2.7%)	4 (6.9%)	
	Other	20 (12.4%)	2 (3.5%)	
Reconstruction site				< 0.001
	Lower jaw	86 (53.4%)	43 (74.1%)	
	Tongue/floor of mouth	31 (19.3%)	7 (12%)	
	Upper jaw	21 (13%)	6 (10.3%)	
	e.o.	10 (6.2%)	—	
	Intermax.	13 (8.1%)	2 (3.6%)	
ND	Yes	79 (49.1%)	27 (46.5%)	NS
Bone resection	Yes	106 (65.8%)	50 (86.2%)	< 0.001
Flap success	Yes	151 (93.8%)	50 (86.2%)	NS
ASA	> 3	124 (78.5%)	48 (84.2%)	NS
HAP	Yes	10 (6.2%)	6 (10.3%)	NS
ICU	Days	4.1 ± 4.9	5.6 ± 6.2	0.003
NW	Days	14.1 ± 7.7	17.6 ± 9.1	< 0.001
AB	Days	13.9 ± 6.5	17.9 ± 6.9	< 0.001
Piperacillin/tazobactam				< 0.001
	Yes	39 (24.2%)	5 (8.6%)	
	No	122 (75.8%)	53 (91.3%)	

Variable		Tumor (*N* = 101)	ORN (*N* = 60)	*p*
Age	Years	66.6 ± 11.1	61.6 ± 8.5	0.04
Flap				< 0.001
	FIB	25 (24.8%)	42 (70%)	
	RFF	35 (34.7%)	6 (10%)	
	ALT	23 (22.8%)	6 (10%)	
	Double	3 (3%)	1 (1.7%)	
	Other	15 (14.7%)	5 (8.3%)	
Reconstruction site				< 0.001
	Lower jaw	34 (33.7%)	53 (88.3%)	
	Tongue/floor of mouth	30 (29.7%)	—	
	Upper jaw	17 (16.8%)	4 (6.7%)	
	e.o.	7 (6.9%)	3 (5%)	
	Intermax.	13 (12.9%)	—	
ASA	> 3	84 (83.2%)	42 (70%)	NS
ICU	Days	4.3 ± 5.2	3.8 ± 4.5	NS
NW	Days	14.7 ± 7.9	13.1 ± 7.1	NS
AB	Days	13.2 ± 6.2	15.2 ± 68	NS
Piperacillin/tazobactam				NS
	Yes	23 (22.8%)	16 (26.7%)	
	No	78 (77.2%)	44 (73.3%)	

Abbreviations: AB: antibiotics; ALT: anterior lateral thigh flap; ASA: Score American Society of Anesthesiologists; e.o.: extraoral; FIB: fibula free flap; intermix.: intermaxillary; ND: neck dissection; NS: not significant; NW: normal ward; RFF: radialis free flap; SSI: surgical site infection.

## 4 Results

### 4.1 Clinical Features

One hundred and sixty‐one patients who underwent microvascular free flap reconstruction after prior radiotherapy between 2016 and 2025 were retrospectively identified and included in the study. Detailed epidemiological and surgical parameters are presented in Table 1.

Among the pre‐irradiated cohort, 63% were tumor patients while 37% were treated for ORN. Overall, 33 patients (20.5%) represented tumor salvage cases (32.6% within tumor cases): 11 following primary radiochemotherapy and 22 with tumor recurrence after adjuvant radiotherapy requiring surgery.

The most frequently used microvascular flaps were the fibula flap (41.6%) and the radial forearm flap (25.5%) (Table 1). In addition, four double flaps were performed: two combinations of fibula + anterolateral thigh (ALT) and two of fibula + radial forearm flap. Under “other” 10 latissimus dorsi flaps, 1 upper arm flap and 9 osseous scapula flaps were subsumed (Table 1). A total of 10 patients (6.2%) developed nosocomial pneumonia (HAP). Descriptive clinical subgroup analysis was performed for tumor and ORN (Table 1). In univariate analysis ORN patients were significantly younger than tumor patients (61.6 ± 8.5 vs. 66.6 ± 11.1 years, *p* = 0.04) and showed different reconstructive patterns with fibula flaps being more common in ORN cases (70% vs. 24.8%) and lower‐jaw reconstructions predominating the ORN cohort (88.3% vs. 33.7%; both *p* < 0.001). In contrast, perioperative factors including ASA > 3 (70% vs. 83.2%), ICU stay (3.8 ± 4.5 vs. 4.3 ± 5.2 days), NW stay (13.1 ± 7.1 vs. 14.7 ± 7.9 days), duration of antibiotic therapy (15.2 ± 6.8 vs. 13.2 ± 6.2 days), and piperacillin/tazobactam allocation did not differ significantly between groups (Table 1).

### 4.2 Antibiosis and SSI

Initially, 39 patients (24.2%) received perioperative broad‐spectrum PAP with piperacillin/tazobactam, whereas the remaining patients were treated with standard prophylaxis: Amoxicillin/clavulanic acid (69.6%), third‐generation cephalosporins (4.4%), and clindamycin (1.9%).

In 58 cases (36%) SSIs were documented. Of these, five cases occurred in patients initially treated with piperacillin/tazobactam (5/39; 12.8%), while 53 cases (53/122; 43.4%) occurred in those who had received standard empiric therapy (*p* < 0.001). Within the piperacillin/tazobactam group, five patients were switched to meropenem based on antimicrobial susceptibility testing and one patient received additional vancomycin. Within the other group, antibiotic prophylaxis maintained as preemptive therapy was escalated to piperacillin/tazobactam (37.7%), ciprofloxacin (24.5%), or both (3.8%). 17% were escalated to meropenem. In three cases, initially calculated therapy with clindamycin was changed to aminopenicillin, or aminopenicillin was augmented with metronidazole. In a total of six patients with SSI, antibiotic therapy was not modified.

In the univariate analysis SSI occurrence showed significant associations with several clinical factors (Table 1). SSI was more frequent in patients with ORN (46.7% vs. 29.7%, *p* = 0.02), in those undergoing fibula flap reconstruction (*p* < 0.001), and in cases involving bone resection (86.2%, *p* < 0.001). Moreover, patients who developed an SSI had a significantly prolonged length of stay both in the intensive care unit (5.6 vs. 4.1 days, *p* = 0.03) and normal ward (17.6 vs. 14.1 days, *p* < 0.001). Salvage surgery showed no significant association with SSI occurrence (30.3% vs. 37.5%; *p* = 0.54).

Among the 122 analyzed cases without initial piperacillin/tazobactam 78 patients (63.9%) were diagnosed with tumor and 44 with ORN (36.1%). In the tumor group, 35.9% developed an SSI requiring escalation, whereas 64.1% did not. Among ORN patients, 56.8% required escalation, while 43.2% did not. Univariate analysis demonstrated a significant association between diagnosis and the occurrence of SSI in this group (Pearson's *χ* ^2^ = 5.011, *p* = 0.025; Fisher's exact test, *p* = 0.036).

Patients who received initial piperacillin/tazobactam demonstrated a significantly lower rate of SSI compared with those treated with standard regimens (12.8% vs. 43.4%, p = < 0.001, Table 2). With respect to antibiotic treatment duration in the piperacillin/tazobactam group, no difference was observed compared to the remainder of the cohort (Table 2). Overall, 33% of patients received therapy for ≤ 10 days, while 50% were treated for 11–21 days, and 17% for > 22 days.

**TABLE 2:** Antibiotic characteristics:

Independent variable	SSI rate	SSI rate	*p*
Piperacillin/tazobactam (no vs. yes)	43.4%	12.8%	< 0.001

Variable	No SSI	SSI	*p*
Antibiotic duration (days)	11.53 ± 4.85	17.9 ± 6.97	< 0.001
Piperacillin/tazobactam yes	13.0 ± 5.9	23.0 ± 6.8	NS
Piperacillin/tazobactam no	11.0 ± 4.3	17.4 ± 6.8	NS

Abbreviation: NS: not significant.

Patients without SSI required a mean treatment duration of 11.4 ± 4.7 days, whereas patients with SSI received significantly longer courses of 17.9 ± 7.0 days (*p* < 0.001). A trend toward prolonged antibiotic therapy was observed in the ORN group compared with the tumor group (15.2 vs. 13.2 days), although this difference did not reach statistical significance (*p* = 0.07). When stratified by SSI status, treatment duration did not differ significantly between ORN and tumor patients without SSI (11.7 vs. 11.5 days, *p* = 0.8).

Under multivariate consideration, bone resection emerged as a strong independent risk factor, being associated with a more than sixfold increased likelihood of SSI occurrence (OR 6.28, *p* < 0.001) with prior exposure to piperacillin/tazobactam showing a significant protective effect, reducing the odds of the event by approximately 87% (OR 0.13, *p* < 0.001).

When stratified by diagnosis, bone resection remained a strong independent risk factor for SSI in both groups, with OR 5.8 (*p* < 0.001) in tumor patients and OR 6.8 (*p* = 0.025) in ORN patients. Prior exposure to piperacillin/tazobactam was associated with a protective effect in both subgroups, reaching statistical significance in the ORN cohort (OR 0.12, *p* < 0.01) and in the tumor cohort (OR 0.13, *p* = 0.01).

### 4.3 Cox Regression Analysis

**FIGURE 1:**
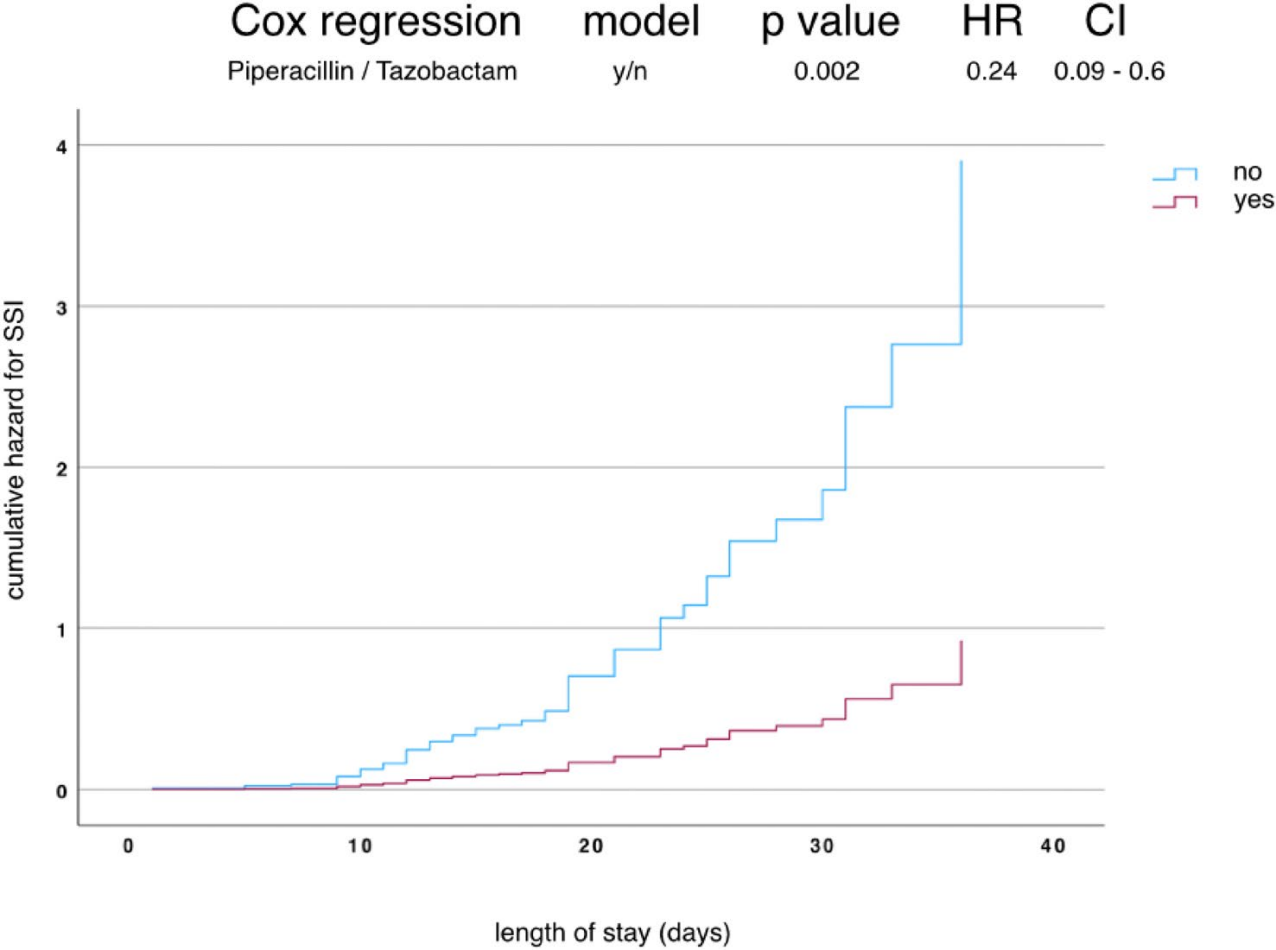
Cox regression analysis and hazard ratio (HR) on overall SSI event separated for piperacillin/tazobactam groups yes/no with 95% confidence intervals (CIs) over length of stay. [Color figure can be viewed at wileyonlinelibrary.com]

In the Cox regression analysis without further subgroup differentiation (Figure 1), calculated exposure to piperacillin/tazobactam was identified as a significant predictor of time to event (SSI). Patients with initial piperacillin/tazobactam treatment exhibited a markedly reduced hazard compared to those without exposure for SSI event (HR = 0.24, 95% CI 0.09–0.6, *p* = 0.002). This corresponds to an approximate 76% reduction in the risk of SSI, indicating a robust protective effect (Figure 1). Combined with bone resection the model showed an even higher protective effect in patients (HR 0.17; CI 0s.041–0.71; *p* = 0.01).

In subgroup analysis, Cox regression, the performed model was statistically significant (*p* < 0.001), indicating that group membership significantly predicted SSI occurrence.

**FIGURE 2:**
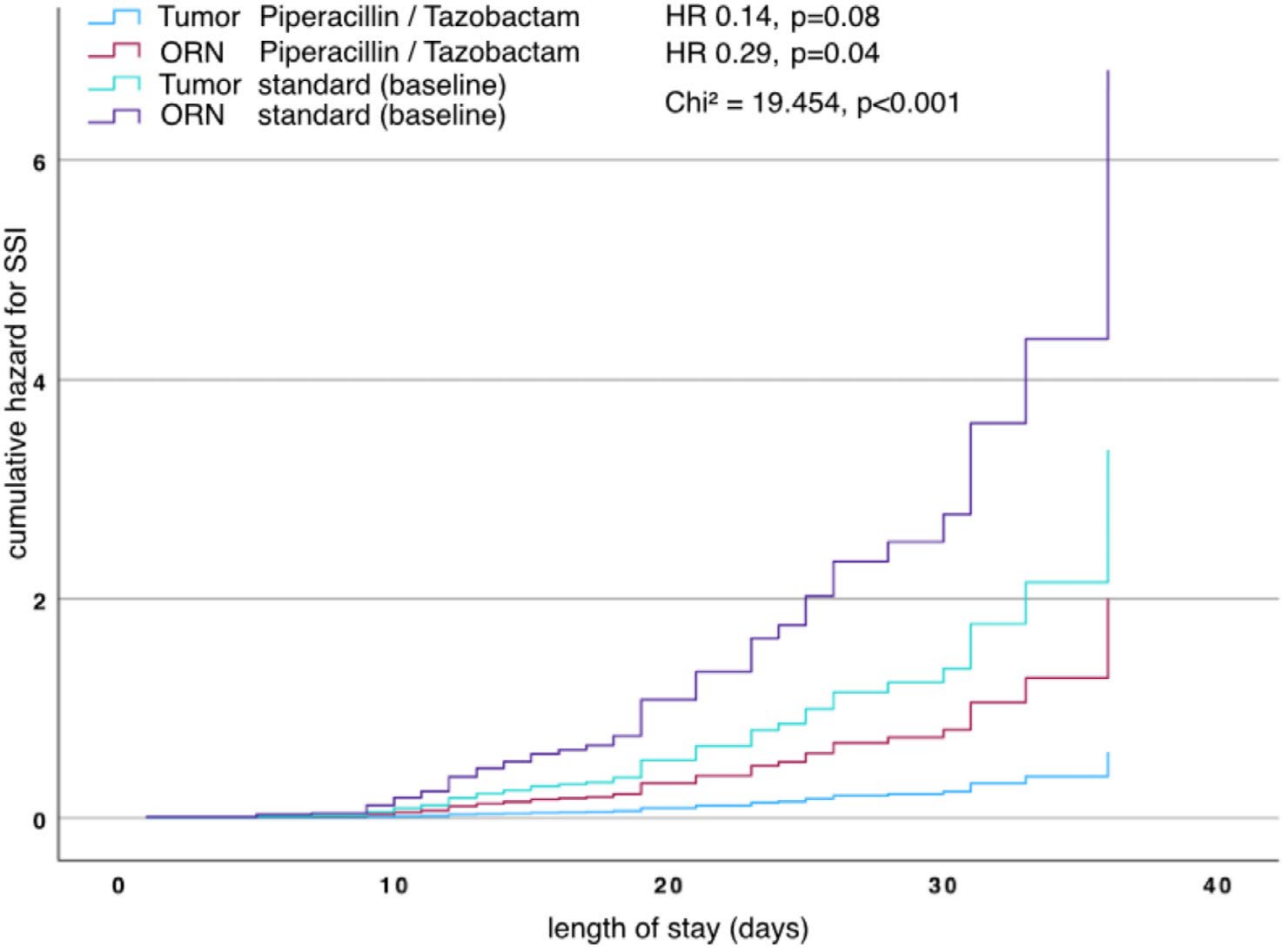
Cox regression analysis comparing hazard ratios (HRs) for subgroup SSI events in ORN and tumor patients with piperacillin/tazobactam to baseline including degrees of freedom and *p*‐values over length of stay. [Color figure can be viewed at wileyonlinelibrary.com]

Using ORN and tumor patients without piperacillin tazobactam as the reference category, tumor patients receiving piperacillin/tazobactam exhibited the strongest reduction in odds of infection (HR 0.14, 95% CI 0.36–0.61, *p* = 0.008), corresponding to an approximate 86% decrease. Similarly, ORN patients with piperacillin/tazobactam had decreased odds compared with the reference group (HR 0.29, 95% CI 0.09–0.98, *p* = 0.04), representing a 71% reduction (Figure 2).

Tumor patients without piperacillin/tazobactam exposure also demonstrated significantly lower odds than ORN without extended‐spectrum coverage (OR 0.49, 95% CI 0.28–0.86, *p* = 0.013).

In patients with SSI and without perioperative piperacillin/tazobactam therapy (*n* = 53) the most frequently isolated microorganism was *Enterobacter cloacae* (30.2%), followed by *Escherichia* *coli* (11.3%). Other relevant isolates included *Streptococci* (7.5%), *Staphylococci* (7.5%), *Klebsiella* spp. (7.5%), and *Candida* spp. (7.5%). Less frequently detected organisms were *Enterococcus* (1.9%), *Pseudomonas* (1.9%), *Serratia* (1.9%), and *Citrobacter* (1.9%). In 20.8% of cases no microbial growth was observed. No growth was observed in one case, while *E. cloacae, Enterococci, Serratia*, and *Klebsiella* spp. were each detected once (see Table 3 and Table 4).

**TABLE 3:** SSI culture results in 58 patients for patients with and without calculated piperacillin/tazobactam.

Without extended spectrum PAP			With extended spectrum PAP		
Total	*N* = 53	100.0%	Total	*N* = 5	100.0%
No pathogen	11	20.8%	No growth	1	20.0%
*Streptococcus* spp.	4	7.5%	*Enterobacter* spp.	1	20.0%
*Staphylococcus* spp.	4	7.5%	*Enterococcus* spp.	1	20.0%
*Enterobacter* spp.	16	30.2%	Serratia	1	20.0%
*Enterococcus* spp.	1	1.9%	*Klebsiella* spp.	1	20.0%
Pseudomonas	1	1.9%			
Serratia	1	1.9%			
*Citrobacter* spp.	1	1.9%			
*Escherichia coli*	6	11.3%			
*Klebsiella* spp.	4	7.5%			
*Candida* spp.	4	7.5%			

**TABLE 4:** SSI culture results in 58 patients separated for tumor and ORN.

Tumor patients			ORN patients		
Total	*N* = 30	100%	Total	*N* = 28	100%
No growth	9	30.0%	No growth	3	10.7%
*Streptococcus* spp.	2	6.7%	*Streptococcus* spp.	2	7.1%
*Staphylococcus* spp.	2	6.7%	*Staphylococcus* spp.	2	7.1%
*Enterobacter* spp.	6	20%	*Enterobacter* spp.	11	39.3%
Pseudomonas	1	3.3%	*Enterococcus* spp.	2	7.1%
Serratia	1	3.3%	Serratia	1	3.6%
*Citrobacter* spp.	1	3.3%	*Escherichia coli*	3	10.7%
*E. coli*	3	10%	Klebsiella spp.	1	3.6%
*Klebsiella* spp.	4	13.3%	*Candida* spp.	3	10.7%
*Candida* spp.	1	3.3%			

## 5 Discussion

To the best of our knowledge, this is the first original study to report on the calculated use of perioperative extended‐spectrum coverage in preirradiated tumor patients and ORN patients undergoing microvascular head–neck reconstruction.

SSI have become the most relevant nosocomial complication after microvascular reconstruction in the head and neck region and pose a substantial challenge to the surgical team, particularly in oncological pretreated patients [24, 25]. Escalation of antibiotic therapy with prolonged treatment courses is frequently necessary, resulting in extended inward occupancy and patients presenting with tumor recurrence, after salvage surgery or reconstruction due to advanced ORN experience a further decline in quality of life [19, 24, 26]. Moreover, prolonged antibiotic exposure disrupts the human microbiome, which is particularly detrimental in the oncological setting where it has been linked to adverse survival outcomes [27, 28]. Therefore, careful selection and standardized implementation of an appropriate perioperative antibiotic regimen to prevent SSI is of utmost importance. It should be emphasized that narrow‐spectrum agents such as penicillin or clindamycin provide insufficient coverage of the mixed gram‐positive and gram‐negative microbiome characteristics of clean‐contaminated head and neck surgery. In this setting, broader‐spectrum agents such as amoxicillin–clavulanic acid or third‐generation cephalosporins are traditionally recommended for perioperative coverage [3, 29].

However, particularly in irradiated patients, the oral microbiome shifts and is frequently colonized by gram‐negative pathogens [14]. In addition, these patients often have a history of repetitive hospitalizations resulting in colonization with multidrug‐resistant organisms [30]. Perioperative suppression of commensal flora combined with radiation‐induced impaired healing and surgical disruption of tissue barriers creates a high‐risk environment for early onset SSI, particularly within the first postoperative week [31, 32, 33]. Most studies advocating shortened perioperative antibiotic coverage are retrospective and limited by preselected cohorts excluding microvascular reconstruction or irradiation [3, 34].

With a ratio of 1:1.7 between ORN and tumor cases in our cohort, ORN patients showed a significantly higher incidence of postoperative SSI than the tumor group (47% vs. 30%, *p* = 0.02) predicting significant prolonged antibiotic treatment times (see Table 1). Bone resection, particularly of the mandible, emerged as a crucial risk factor for infection reaching multivariate significance not only in the ORN but also in the tumor cohort (*p* < 0.001). With an overall SSI incidence of 36% in the study cohort, our findings fall within the findings of Cannon et al. suggesting a “high‐risk” SSI profile for irradiated patients [1]. Although salvage tumor procedures with rescue flaps after failed microvascular reconstruction have been associated with particularly high SSI rates, this was not observed in our cohort, where salvage surgery showed no increased risk for SSI occurrence (*p* = 0.5) [35].

Calculated initial perioperative coverage with an extended‐spectrum antibiosis demonstrated a statistically significant benefit with a marked four‐fold risk reduction in SSI risk across the entire cohort. This effect remained robust in both diagnostic subgroups and for patients with intraoperative bone resection after multivariate stratification. To date, this has not been reported or suggested in the literature as no study design has systematically applied extended‐spectrum perioperative antibiotic coverage in microvascular head–neck reconstruction.

In abdominal surgery D' Angelica et al. showed in a multicenter randomized clinical trial that patients undergoing open pancreatoduodenectomy had significantly reduced 30‐day SSI rates if initially covered with piperacillin/tazobactam [36]. Given comparably high baseline SSI rates (≥ 30%), the anatomical complexity of the surgery and the lack of quality‐of‐life–preserving alternatives, this clinical approach can be reasonably extrapolated to patients with microvascular head–neck reconstructions after radiotherapy. As an empiric option, piperacillin/tazobactam covers *Pseudomonas aeruginosa* , many Enterobacterales, anaerobes, and *Enterococcus faecalis* , and pharmacokinetic data show therapeutically relevant jaw‐bone concentrations in in vivo studies [37, 38].

In ORN surgery intraoperative cultures frequently yield gram‐negative bacilli with notable multidrug resistance, whereas gram‐positive cocci are often broadly susceptible to standard penicillin regimen [30, 39]. Regarding cultivated pathogens in this study, we observed in the SSI group a trend toward gram‐negative bacteria with intrinsic resistance to narrow‐spectrum beta‐lactam antibiotics. Most notably, the *Enterobacter* spp. frequently producing extended‐spectrum β‐lactamases was more prevalent in the ORN cohort leading to antibiotic escalation (20% vs. 39%). As Arianpour et al. stated, besides changes in the oral microbiome through structural changes, this finding is likely attributable to the extensive prior oral antibiotic exposure in this patient population, which confers a selective advantage to resistant strains [39].

With 57% in subgroup analysis ORN without initial extended spectrum coverage showed the highest incidence and HR for SSI event. Cox regression model showed a significant sevenfold reduction in tumor and a threefold reduction ORN with extended‐spectrum antibiotic coverage. In our analysis, irradiated tumor patients receiving broad‐spectrum coverage showed a sevenfold reduction in SSI risk without prolonging baseline antibiotic administration times while ORN patients showed a threefold risk reduction. This difference may be explained by the underlying microbiological features. While classical studies on microbiological profiles of SSI in tumor patients typically describe polymicrobial infections with a predominance of gram‐negative species, these pathogens are often susceptible to standard antibiotic regimens [40]. In contrast, ORN is characterized by a higher incidence of multidrug‐resistant gram negative and positive strains, with beta lactam resistance rates exceeding 50% [39]. As already mentioned, patients with ORN often present with bone to skin fistula as first clinical manifestation which frequently results in prolonged administration of oral antibiotics, further complicating management and potentially contributing to the selection of resistant pathogens [41]. Notably, Arianpour et al. have suggested that adapted to *Staphylococcus aureus* isolates the microbial milieu in ORN may even warrant an initial regimen combining vancomycin plus piperacillin/tazobactam to achieve adequate broad‐spectrum coverage [39].

Williams et al. recently described a significant reduction in SSI prevalence in an ORN cohort reconstructed with both microvascular and pedicled flaps, achieved through intraoperative sampling and adapted postoperative culture‐directed therapy [13]. Notably, this study does neither provide detail to determine the frequency and quality of antibiotic escalation nor the proportion of cases in which calculated broad‐spectrum therapy was maintained. Several studies, employing both culture‐based and metagenomic approaches, have demonstrated that predictive perioperative cultures are not reliable for anticipating SSI in tumor patients [42]. Prior irradiation leads to fibrosis and capillary damage, resulting in markedly delayed wound healing, which secondarily predisposes to infections frequently caused by gram‐negative pathogens [30, 43, 44].

Argawal et al. constitutes that in the context of ORN—where management is typically initiated with antibiotic therapy—intraoperative biopsies from necrotic bone may fail predicting SSI [18]. Nevertheless, intraoperative cultures should be seen as the gold standard for guiding empirical postoperative antibiotic therapy and should be incorporated into an ABS algorithm.

Our data suggests that in both irradiated tumor and ORN patients calculated piperacillin/tazobactam coverage is a pragmatic strategy to prevent early‐onset SSI. It is important to emphasize the impact of this calculated intervention in previously irradiated tumor patients. In this context, a postoperative compromise of microvascular reconstruction may not only impair quality of life in a salvage setting but also decisively affect the patient's overall survival. Early‐onset SSIs, fistula formation, and partial necrosis prolong hospitalization and increase the incidence of reintubation and hospital‐acquired pneumonia [45, 46, 47]. Persistent inflammatory states further promote tumor recurrence and prolonged antibiotic treatment may further disrupt bacterial eubiosis, impairing results of adjuvant immunotherapy [27, 48].

This study has several limitations to consider. The findings are restricted to microvascular reconstructions and are not directly comparable to smaller surgical procedures in irradiated patients. In addition, the retrospective design limits the strength of clinical recommendations regarding extended‐spectrum perioperative coverage in patients after radiotherapy. The microbiological cultures in this study were obtained postoperatively in cases of SSI, which may limit their diagnostic reliability. Future investigation should be conducted in a prospective controlled setting to confirm these results. Particular emphasis should be placed on the implementation of ABS principles. Intraoperative cultures, especially in patients with ORN, are the cornerstone for culture‐guided postoperative escalation or de‐escalation managing postoperative SSI.

## 6 Conclusion

Patients with a history of head and neck irradiation and bone resection represent a high‐risk cohort, with an incidence of early‐onset SSI exceeding 30% and significant prolonged courses of antibiotic therapy. Overall, this patient group benefits significantly from perioperative extended‐spectrum antibiotic coverage with piperacillin/tazobactam showing four to fivefold SSI risk reduction. Subgroup analyses confirmed a threefold protective effect in ORN and a sevenfold reduced risk in tumor patients. An initial calculated broad‐spectrum coverage in preirradiated patients undergoing microvascular reconstruction may substantially lower the risk of early‐onset SSI.

## Funding

The authors have nothing to report.

## Ethics Statement

Approved by the local Ethics Committee (Nr: 18‐1131‐104).

## Consent

The authors have nothing to report.

## Conflicts of Interest

The authors declare no conflicts of interest.

## Data Availability

The data that support the findings of this study are available on request from the corresponding author. The data are not publicly available due to privacy or ethical restrictions.
